# Systematic analysis of changes in cannabis use among participants in control conditions of randomised controlled trials

**DOI:** 10.1016/j.abrep.2015.06.001

**Published:** 2015-06-05

**Authors:** Shane Rebgetz, Leanne Hides, David J. Kavanagh

**Affiliations:** a Institute of Health & Biomedical Innovation and School of Psychology & Counselling, Queensland University of Technology, Brisbane, QLD, Australia; b Queensland Health, Metro North Hospital and Health Service, Redcliffe-Caboolture Mental Health Service, QLD, Australia

**Keywords:** Cannabis, Self-management, Natural recovery, Control conditions

## Abstract

**Introduction:**

Cannabis remains the most used illegal substance across the globe, and negative outcomes and disorders are common. A spotlight therefore falls on reductions in cannabis use in people with cannabis use disorder. Current estimates of unassisted cessation or reduction in cannabis use rely on community surveys, and few studies focus on individuals with disorder. A key interest of services and researchers is to estimate effect size of reductions in consumption among treatment seekers who do not obtain treatment. Effects within waiting list or information-only control conditions of randomised controlled trials offer an opportunity to study this question.

**Method:**

This paper examines the extent of reductions in days of cannabis use in the control groups of randomised controlled trials on treatment of cannabis use disorders. A systematic literature search was performed to identify trials that reported days of cannabis use in the previous 30 (or equivalent).

**Results:**

Since all but one of the eight identified studies had delayed treatment controls, results could only be summarised across 2–4 months. Average weighted days of use in the previous 30 days fell from 24.5 to 19.9, and a meta-analysis using a random effects model showed an average reduction of 0.442 SD. However, every study had at least one significant methodological issue.

**Conclusions:**

While further high-quality data is needed to confirm the observed effects, these results provide a baseline from which researchers and practitioners can estimate the extent of change required to detect effects of cannabis treatments in services or treatment trials.

## Highlights

• Systematic searches identified 8 trials reporting days of cannabis use per month in delayed or information-only controls
• Sample-weighted mean days of use fell from 24.5 per month at Baseline to 19.9 at 2-4 months
• A meta-analysis showed an average reduction of 0.442 SD.
• While there was substantial continued use, this reduction sets a challenging benchmark for treatments.

## 1 Introduction

Cannabis remains the most used illegal drug across the world, and while rates of use are generally falling, the incidence of related harm is rising internationally (United Nations Office on Drugs and Crime, 2014). Australia has particularly high rates of use, with 35% of adults reporting lifetime consumption, and 10% using it in the previous 12 months (Australian Institute of Health and Welfare, 2014).

However, 70–80% of cannabis users stop using it by their mid-thirties (Chen & Kandel, 1998), and even over 5–6 years, substantial rates of cessation or reduced consumption in adolescents or young adults are seen (Kandel and Raveis, 1989, Pollard et al., 2014, Sussman and Dent, 2004). In common with other substances, most successful cessation occurs without treatment (Cunningham, 2000, Price et al., 2001). While these changes are typically greatest among infrequent or non-problematic users (Chen & Kandel, 1998), people with cannabis abuse or dependence also have substantial rates of recovery. For example, an analysis of data from Wave 1 of the National Epidemiologic Survey on Alcohol and Related Conditions (Agosti & Levin, 2007) found that 81% of people with lifetime cannabis dependence did not meet criteria over the previous year.

While community samples can provide good estimates of the degree and timing of recovery from cannabis use disorder, sample sizes need to be large to provide accurate estimates of these rates. So, a study of 1228 adolescents (Perkonnigg et al., 1999) found only 12 with lifetime cannabis dependence, and the resultant estimate of full remission (32%) therefore had a substantial standard error (26%). Furthermore, treatment trial researchers and services need estimates of remission in treatment seekers.

A study of control groups in treatment studies provides fertile ground for the estimation of changes in treatment seekers who do not receive substantial assistance. These studies have several advantages: high-quality trials typically have diagnostic interviews and other assessments that are able to characterise the samples well, the nature of treatments is standardised or tracked carefully, and substantial effort is put into ensuring that follow-up assessments maximise retention rates. While individual studies often have relatively small sample sizes in their control group, meta-analytic methods provide an opportunity to obtain estimations of effect sizes over multiple studies and substantial samples.

Accordingly, the aim of the current paper was to determine the degree of ‘natural recovery’ in the control groups from randomised controlled trials on substance use disorders, which reported changes in the frequency of cannabis use. ‘Natural recovery’ in this article refers to processes where consumption of cannabis is reduced or ceased without professional intervention. It was operationalised as the degree of change in cannabis use within groups receiving inactive or minimal interventions.

## 2 Methods

Electronic searches were performed in January 2015, to find studies that included a control group that had explored the topic of cannabis use treatment. The search used title, abstract and keywords of Medline, PsycINFO, Psychology Journals, and Psychology Subject Corner. The search terms were: (cannabis OR marijuana OR marihuana OR addiction OR abuse OR substance) AND (treatment OR randomi* control).

Potential studies were evaluated for inclusion in this study by the first author, based on whether they: (a) provided data on cannabis use, which allowed the calculation of pre–post effect sizes in a group of participants randomised to receive inactive (e.g. waitlist) or minimal interventions (e.g. drug-related information only); (b) were in English; (c) did not comprise case studies or personal accounts; (d) did not include participants with severe mental disorders (i.e., schizophrenia, bipolar disorder, posttraumatic stress disorder, major depressive disorder). In order to report results on a single measure, we restricted the studies to those allowing a calculation of cannabis use in the previous 30 days.

The formal examination of effect sizes used Comprehensive Meta-Analysis (Borenstein, Hedges, Higgins, & Rothstein, 2005), and the primary analysis applied a random effects model. This is the appropriate approach to use when samples or treatments are potentially different, regardless of whether significant heterogeneity is evidenced (Borenstein, Hedges, Higgins, & Rothstein, 2009). We report effects as standardised mean differences (Cohen's d). Analyses of degree of change require estimates of test–retest correlations of the measures, or reported analyses of changes within groups. While Timeline Followback assessments of cannabis use can have a 7–14 day test–retest reliability of 0.92 (Robinson, Sobell, Sobell, & Leo, 2014), we do not know the reliability of the 3–12 month assessments of cannabis use in the current trials. We use an estimate of 0.70 for the primary analyses below, but also undertake sensitivity analyses with test–retest correlations of .60 and .80. Where means and standard deviations were reported on different sample sizes at baseline and follow-up, we used the follow-up sample size for the analysis, estimating baseline scores for retained participants from reported data using the full sample. We also present sample-weighted mean days of use at baseline, post and follow-up assessments.

## 3 Results

The search of cannabis treatment in general population samples elicited 2554 articles. Reviewing article titles to confirm that they met the search criteria left 374, and this number was reduced to 55 after reading abstracts. Further searching using reference lists and cited reference search yielded 12 potential articles, and 3 others were suggested by reviewers. Review papers were examined (Carballo et al., 2007, Dutra et al., 2008, Sobell et al., 2000, Tanner-Smith et al., 2013) to identify any additional papers, but none were added from that procedure. A final decision on inclusion was determined after reading the full paper, and any that raised potential questions on inclusion were reviewed by all authors, until consensus was reached. Studies by Copeland et al. (2001), Lozano et al. (2006), Kadden et al. (2007), Kay-Lambkin et al. (2009), Fernandes et al. (2010), Peters et al. (2011), Stein et al. (2011), Walker et al. (2011), Litt et al. (2013) and Hoch et al. (2014) were excluded due to an inability to calculate a within-group effect size on cannabis use per month from the data provided. The control groups of Stephens et al. (1994), Hendriks et al. (2011) and Budney et al. (2000) provided too much support for them to meet inclusion criteria as a control treatment condition.

Details of the eight included studies are displayed in Table 1, their results are provided in Table 2 and their methodological quality is summarized in Table 3. The studies had a total of 600 control participants. Average weighted mean days of use in the previous 30 days fell from 24.5 to 19.9 at 2–4 months across the eight studies. Only one of the included studies (Fischer et al., 2012) provided data over a longer follow-up, preventing an assessment of the degree of sustained change across the studies. That study saw little change in use at 12 months (M = 22.1, SD = 9.2).

**Table 1:** Studies on treatment of cannabis use in the past 30 days within control groups of general populations: Studies reporting mean values.

Author (date)	Sample type	Basis of participation	Disorder	Country	Control group	Measure
Stephens et al. (2000)	COM	Wanting help quitting	98% current CUD	US	Delayed treatment	# days used cannabis per month
Litt et al. (2005)	COM	Treatment	100% current CUD	US	Delayed treatment	% days used cannabis in the past 90
Walker et al. (2006)	SCH	Information re their CU	68% current CUD (86% lifetime CUD)	US	Delayed treatment	# days used cannabis in the past 60
Stephens et al. (2007)	COM	Feedback on CU (not treatment)	93% current CUD	US	Delayed feedback	# days used cannabis per week
Martin and Copeland (2008)	COM + OP	Information, discussion	85% CUD	AU	Delayed treatment	# days used cannabis in the past 90
Fischer et al. (2012)	UNI	…^1^	CU	CAN	General health information	# days used cannabis in the past 30
Gates et al. (2012)	COM	Information or counselling on CU concerns	98% probable CUD on SDS	AU	Delayed treatment	# days used cannabis in the past 28
Rooke et al. (2013)	COM	Wanting to reduce or cease CU	CU	AU	Cannabis information	# days used cannabis in past month

AU: Australia; CAN: Canada; US: United States of America;

OP: Outpatients; COM: Community; HM: Homeless/unstably housed; SCH: School; UNI: University;

CU: Cannabis use; CUD: Cannabis use disorder (DSM-IIR or DSM-IV Cannabis Dependence or Abuse);

SDS: Severity of Dependence Scale (Gossop et al., 1992).

1 Mass advertising described the intervention study. Specific details on the basis of participation are not provided.

**Table 2:** Mean days of cannabis use in the past 30 days, in control groups of treatment trials on people with cannabis use disorders.

Study	Baseline			2–4 months		
	N	M	SD	N	M	SD
Stephens et al. (2000)	86	24.9	6.1	79	17.1	10.7
Litt et al. (2005)^1^	148	30.0	4.7	148	25.2	10.2
Walker et al. (2006)^2^	50	18.4	8.5	50	16.4	10.3
Stephens et al. (2007)^3^	64	26.0	8.2	64	24.6	8.2
Martin and Copeland (2008)^4^	20	18.5	10.5	20	18.2	10.5
Fischer et al. (2012)	32	23.9	6.1	32	23.1	6.9
Gates et al. (2012)^5^	81	23.9	6.3	61	13.4	12.2
Rooke et al. (2013)	119	20.8	8.7	58	14.1	8.8
Total N, weighted mean	600	24.5		512	19.9	

Conversion formulae from reported means (M) to give days of use in the past 30 days:.

1 % days used in past 90: M × 30.

2 Days used in past 60: M/2.

3 Days per week: (M/7) × 30.

4 Days used in past 90: M/3.

5 Days used in past 28: (M/28) × 30.

**Table 3:** Methodological review of control treatments from the included randomised controlled trials.

Study	Symptom/diagnostic measure	Treatment received by controls	Follow-up retention	Intention to treat (and management of missing data)	Single-blind follow-up
Stephens et al. (2000)	CUD: Self-report CU: Self & collateral report	BL: No current formal treatment 4 mths: 6% had treatment 18% in self-help groups	92% to 4 mths	No	No—Self-report (phone interview if no response)
Litt et al. (2005)	CUD: SCID CU: TLFB, Self & collateral report, urinalysis.	BL: No current Mj therapy, self-help group 4 mths: NR	93% to 4 mths	No (Secondary analyses: BL substitution)	No
Walker et al. (2006)	CUD: GAIN CU: Self-report	NR	98% to 3 mths	No	NR—Self-report; different staff at follow-up
Stephens et al. (2007)	CUD: SCID CU: TLFB, self-report, urinalysis.	BL: No current Mj therapy, self-help group At 7-wks, 6 & 12 mths: 1–4% of whole sample in treatment 2–7% in self-help groups	97% at 7 wks	Yes (BL substitution. Checked with imputation, omission)	No
Martin and Copeland (2008)	CUD: Structured interview (GAIN) & self-report (SDS) CU: TLFB, self-report, urinalysis.	BL: No treatment in previous 90 days 3 mths: NR	80% at 3 mths	Yes (BL substitution)	NR (Independent researcher)
Fischer et al. (2012)	CU: Interviewer-administered questionnaire	NR	52% at 12 mths	No Analysed completers of all assessments	NR
Gates et al. (2012)	Probable CUD: SDS CU: TLFB, self-report.	BL: No current Mj therapy 3 mths: 46% sought treatment, 39% used medication	75% at 3 mths	Yes (Multiple imputation)	No
Rooke et al. (2013)	CUD: GAIN CU: TLFB, Self-report	BL: No formal Mj treatment in last 3 mths 3 mths: Excluded 4% who had treatment	66% at 6 wks 52% at 3 mths	No (Complier average causal effect analyses. Checked with LOCF, omission)	Automated self-report

CU: Cannabis use; CUD: Cannabis Use Disorder;

SCID: Structured Interview for DSM-IV; GAIN: Global Appraisal of Individual Needs (Initial or final) (Dennis, 1998, Dennis, 1999);

TLFB: Timeline Follow-Back; SDS: Severity of Dependence Scale (Gossop et al., 1992);

LOCF: Last observation carried forward; BL: Baseline; Mj: Marijuana; NR: Not Reported.

**Fig. 1:**
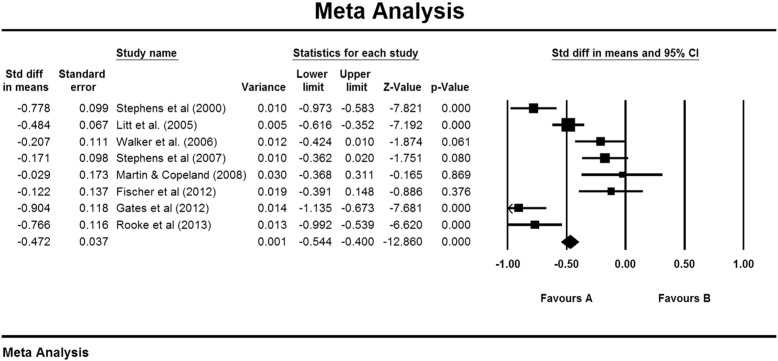
Control group changes over 2–4 months in non-psychotic groups.

Results of the meta-analysis using a test–retest correlation of .70 are displayed in Fig. 1. The figure displays the average effect using a fixed-effects model. With a random effects model, there was an average change in cannabis use of −.442 SD (CI: −.657 to −.228), which was highly significant (*p* < .001). A test of heterogeneity gave Q (7) = 57.71, *p* < .001, providing support for the selection of the random effects model. Examination of the classic fail-safe N found that 293 missing studies would be required to give *p* > .05. Sensitivity analyses using random-effects models and test–retest correlations of .60 (−.460, CI: −.685 to −.235) and .80 (−.415, CI: −.613 to −.217) made little difference to the result.

Four studies specifically targeted people who wanted to quit or reduce cannabis use, or recruited them for an intervention trial (Table 1). The remainder recruited people for information or discussion about their cannabis use. Inspection of Fig. 1 shows that the latter group included three of the four studies with weaker effects (Martin and Copeland, 2008, Stephens et al., 2007, Walker et al., 2006), but it also included the trial with the strongest effects (Gates et al., 2012).

An evaluation of the methodological quality of the control group data is in Table 2. A strength of the studies was their follow-up rates over the control period, with six having rates of 75% or above and four having rates above 90%. None clearly had single-blind follow-up, but two studies had an independent assessor conducting the follow-up, and three used only self-report. Four studies checked participant reports of cannabis use during follow-up against collateral data or urinalysis. All but two studies verified that most participants had a cannabis use disorder, although only two used a gold-standard structured clinical interview. A significant potential threat to the interpretation of results as being reflective of unassisted recovery was the lack of reports on other concurrent treatment in four trials, and a high level of reported treatment in one (Gates et al., 2012). Every study had at least one significant issue that should induce caution in the interpretation of its results.

## 4 Discussion

Control groups from the eight randomised controlled trials showed a significant mean reduction in days of cannabis use. At 2–4 months' follow up, participants used cannabis on 4.6 fewer days a month than at baseline, reflecting over one additional day of abstinence each week, and giving a total of more than a week of total abstinence each month. The average effect size of −.415 to −.442 SD offers a challenging base from which treatment effects are to be obtained. Our results will assist in minimum sample size calculations for randomised controlled trials, and provide a yardstick for the evaluation of changes from services for cannabis misuse.

While we regard these self-initiated changes as substantial, they fall short of major changes in sustained cessation, which supports the contention that self-initiated cannabis cessation is difficult. This observation is consistent with a comparison of reviews on placebo interventions for different substances by Moore and Aubin (2012), which found that nicotine provided the lowest abstinence rate (8%), followed by cannabis (15%) and alcohol (18%), with opioids (44%) and cocaine (47%) providing the strongest responses. While the meta-review potentially resulted in multiple counting of trials and there were few cannabis trials in a single included review, the study highlighted the limited nature of cannabis cessation rates, even in placebo conditions.

Interpretation of our results must be moderated by the issues raised in our methodological review of the studies, which identified at least one significant limitation in every study. Perhaps most important was the potential for other treatment to have been responsible for at least some of the observed reductions in cannabis use. The results highlight areas for future improvement of randomised controlled trials on cannabis use disorder that will not only provide increased confidence in the estimates of change in control groups, but also in the reported outcomes of the whole trial.

While there has been research into unassisted cessation of substance misuse for more than 40 years (Carballo et al., 2007, Sobell et al., 2000), it is only in the last 15 that this work has focused specifically on cannabis. To our knowledge, the current review is the first to examine ‘natural recovery’ in the control groups of randomised controlled trials. Regression to the mean may account for some of the observed change, but our results are consistent with population studies (Agosti and Levin, 2007, Perkonigg et al., 1999), which have similarly observed the potential for recovery from both cannabis use and cannabis dependence, suggesting that at least some individuals can reduce their cannabis use without significant help.

A limitation of this review was the fact that the initial literature search relied on one author, although the resolution of any identified issues and final decisions on inclusion were by consensus of all authors, and no additional papers were identified from reviews. Other limitations included the small number of identified trials with control groups that had no or minimal treatment, and the fact that minimal treatment controls can typically be conducted for periods of only 2–4 months at most. We excluded eight studies because of an absence of data on cannabis consumption over a specific period, in order to preserve comparability of the results across studies: if those studies had provided consumption data, we could potentially have doubled the number of studies in our review. We recommend that future studies routinely include both abstinence rates and average consumption data as part of their results (Peters et al., 2011). However, despite the restricted number of studies, the total sample size of 600 provided a substantial group for estimation of consumption changes.

## 5 Conclusions

This is the first meta-analysis to explore changes in cannabis use in control conditions of treatment studies. Results of the current study demonstrate that modest average reductions in the frequency of average cannabis use can be seen, although there was substantial variability in effect size between studies, and some uncertainty over the potential role of outside treatment in the effects. The study gives weight to further exploration of the concept of natural recovery in people with cannabis use disorders and provides researchers and practitioners a baseline from which to estimate likely changes or needed effects sizes in intervention studies.
